# Anticancer and apoptotic activities of oleanolic acid are mediated through cell cycle arrest and disruption of mitochondrial membrane potential in HepG2 human hepatocellular carcinoma cells

**DOI:** 10.3892/mmr.2021.12079

**Published:** 2021-04-12

**Authors:** Yue-Yong Zhu, Hong-Yan Huang, Yin-Lian Wu

Mol Med Rep 12: 5012-5018, 2015; DOI: 10.3892/mmr.2015.4033

Following the publication of the above paper, a concerned reader drew to the Editor's attention that several figures (principally, Figs. 3, 6 and 8) contained data that bore striking similarities to data published in other papers, some of which had been published around the same time and written by different authors based at different research institutions.

After having conducted an independent investigation in the Editorial Office, the Editor of *Molecular Medicine Reports* has determined that this article should be retracted from the Journal on account of a lack of confidence concerning the originality and the authenticity of the data. The authors were asked for an explanation to account for these concerns, but the Editorial Office never received any reply. The Editor regrets any inconvenience that has been caused to the readership of the Journal.

